# Supplementary material for: Gammaherpesvirus-infected germinal center cells express a distinct immunoglobulin repertoire
Source: Life Sci Alliance. 2020 Feb 6;3(3):e201900526. doi: 10.26508/lsa.201900526 (PMC7012147; doi:10.26508/lsa.201900526)
Supplement: Supplementary file 1 [file LSA-2019-00526_TableS1.docx]

|  | **Sample 1** | | **Sample 2** | | **Sample 3** | | **Sample 4** | | **Sample 5** | | **Total** | |
| --- | --- | --- | --- | --- | --- | --- | --- | --- | --- | --- | --- | --- |
|  | **MHV+** | **MHV-** | **MHV+** | **MHV-** | **MHV+** | **MHV-** | **MHV+** | **MHV-** | **MHV+** | **MHV-** | **MHV+** | **MHV-** |
| Number of sorted cells | 368 | 368 | 207 | 184 | 184 | 184 | 90 | 90 | 90 | 90 | 939 | 916 |
| Number of V_H_ amplicons | 163 | 140 | 74 | 87 | 76 | 90 | 76 | 64 | 51 | 62 | 440^a^ | 443^b^ |
| Number of V_K_ amplicons | 77 | 137 | 72 | 60 | 39 | 87 | 30 | 42 | 38 | 73 | 256 | 399 |
| Number of V_L_ amplicons | 64 | 20 | 48 | 34 | 68 | 16 | 54 | 16 | 35 | 5 | 269 | 91 |
| Number of cells with V_H_ and V_K_ | 38 | 68 | 33 | 33 | 16 | 51 | 12 | 30 | 17 | 44 | 116 | 226 |
| Number of cells with V_H_ and V_L_ | 38 | 7 | 19 | 12 | 32 | 7 | 22 | 11 | 16 | 3 | 127 | 40 |
| Number of cells with V_H_, V_K_ and V_L_ | 4 | 5 | 1 | 8 | 9 | 8 | 7 | 2 | 7 | 2 | 28 | 25 |

V_H_ – immunoglobulin heavy chain variable region

V_K_ – immunoglobulin kappa light chain variable region

V_L_ – immunoglobulin lambda light chain variable region

^a^ number of high quality (average quality >30) productive MHV68^+^ IgH sequences with annotated V, D and J genes used for analysis in this paper: 329

^b^ number of high quality (average quality >30) productive MHV68^-^ IgH sequences with annotated V, D and J genes used for analysis in this paper: 360

Table S1
