# Supplementary material for: Gammaherpesvirus-infected germinal center cells express a distinct immunoglobulin repertoire
Source: Life Sci Alliance. 2020 Feb 6;3(3):e201900526. doi: 10.26508/lsa.201900526 (PMC7012147; doi:10.26508/lsa.201900526)
Supplement: Supplementary file 2 [file LSA-2019-00526_TableS2.docx]

|  | Germinal center cell population | Number of unique reads | Number of productive reads | Number of initial clusters | Number of clonal groups | Number of clonal groups bigger than 1 | Number of overlapping clones |
| --- | --- | --- | --- | --- | --- | --- | --- |
| Animal 1 | MHV+ | 639 064 | 531 391 | 970 | 15 352 | 4 429 | 743 |
|  | MHV- | 527 530 | 346 418 | 1 625 | 21 026 | 7 623 |  |
| Animal 2 | MHV+ | 277 634 | 166 917 | 766 | 6 691 | 2 128 | 772 |
|  | MHV- | 388 196 | 290 651 | 1 502 | 17 366 | 6 449 |  |
| Animal 3 | MHV+ | 384 504 | 236 192 | 655 | 8 191 | 2 358 | 588 |
|  | MHV- | 531 516 | 363 863 | 1 682 | 19 580 | 6 872 |  |

Table S2.
