# Supplementary material for: Gammaherpesvirus-infected germinal center cells express a distinct immunoglobulin repertoire
Source: Life Sci Alliance. 2020 Feb 6;3(3):e201900526. doi: 10.26508/lsa.201900526 (PMC7012147; doi:10.26508/lsa.201900526)
Supplement: Supplementary file 3 [file LSA-2019-00526_TableS3.docx]

|  | **Primer name** | **Primer sequence ^a^ (5’-3’)** | **Reference** |
| --- | --- | --- | --- |
| cDNA synthesis | 3’Cμ_out  3’Cγ_out  3’C_ κ  3’mCλ out | AGGGGGCTCTCGCAGGAGACGAGG  GGAAGGTGTGCACACCGCTGGAC  GTGCTGTCTTTGCTGTCCTGAT  GTACCATYTGCCTTCCAGKCCACT | (Tiller, Busse et al. 2009)  (Tiller, Busse et al. 2009) |
| IgH 1^st^ PCR | 5’MsVHE  3’Cμ_out  3’Cγ_out | GGGAATTCGAGGTGCAGCTGCAGGAGTCTGG  AGGGGGCTCTCGCAGGAGACGAGG  GGAAGGTGTGCACACCGCTGGAC | (Tiller, Busse et al. 2009)  (Tiller, Busse et al. 2009)  (Tiller, Busse et al. 2009) |
| IgH 2^nd^ PCR | 5’MsVHE  3’Cμ_in  3’Cγ_in | GGGAATTCGAGGTGCAGCTGCAGGAGTCTGG  AGGGGGAAGACATTTGGGAAGGAC  GCTCAGGGAAATAGCCCTTGAC | (Tiller, Busse et al. 2009)  (Tiller, Busse et al. 2009)  (Tiller, Busse et al. 2009) |
| IgK 1^st^ PCR | 5’Vk1,2F  5’Vk4F  5’Vk6F  5’Vk8F  5’Vk12F  5’Vk19F  5’L-Vk_3  5’L-Vk_5  5’L-Vk_6,8,9  5’L-Vk_14  5’L-Vk_20  3’B_κ | GGTGCTGATGYTCTGGAT  CAGCTTCCTGCTAATCAGTG  TGCTCTGGTTGTMTGGTG  CTGCTATGGGTRTCTGGT  TGTTGCTGTGGCTTACAG  GGGGCTCTTGTTGYTCTG  TGCTGCTGCTCTGGGTTCCAG  TTTTGCTTTTCTGGATTYCAG  ATGGAATCACAGRCYCWGGT  TCTTGTTGCTCTGGTTYCCAG  CTCACTAGCTCTTCTCCTC  TTCAGGACGCCATTTTGTCG | (Tiller, Busse et al. 2009)  (Tiller, Busse et al. 2009)  (Tiller, Busse et al. 2009)  (Tiller, Busse et al. 2009)  (Tiller, Busse et al. 2009) |
| IgK 2^nd^ PCR | 5’mVκ  3’mCk | GAYATTGTGMTSACMCARWCTMCA  GATGGTGGGAAGATGGATACAGTT | (Tiller, Busse et al. 2009)  (Tiller, Busse et al. 2009) |
| IgL 1^st^ PCR | 5’mVλ1/2  5’mVλx  3’mCλ out | CAGGCTGTTGTGACTCAG  CAACTTGTGCTCACTCAG  GTACCATYTGCCTTCCAGKCCACT | (Tiller, Busse et al. 2009)  (Tiller, Busse et al. 2009)  (Tiller, Busse et al. 2009) |
| IgL 2^nd^ PCR | 5’mVλ1/2  5’mVλx  3’mCλ inn | CAGGCTGTTGTGACTCAG  CAACTTGTGCTCACTCAG  CTCYTCAGRGGAAGGTGGRAACA | (Tiller, Busse et al. 2009)  (Tiller, Busse et al. 2009)  (Tiller, Busse et al. 2009) |

^a^According to The International Union of Pure and Applied Chemistry (IUPAC): K = G or T, Y = C or T, R = A or G, M = A or C, S = G or C, W = A or T.

Tiller, T., C. E. Busse and H. Wardemann (2009). "Cloning and expression of murine Ig genes from single B cells." J Immunol Methods **350**(1-2): 183-193.

Table S3
